# The Myth of Median Nerve in Forearm and Its Role in Double Crush Syndrome: A Cadaveric Study

**DOI:** 10.3389/fsurg.2021.648779

**Published:** 2021-09-21

**Authors:** Sahar A. Abdalbary, Mohamed Abdel-Wahed, Sherif Amr, Mostafa Mahmoud, Ehab A. A. El-Shaarawy, Safinaz Salaheldin, Amal Fares

**Affiliations:** ^1^Department of Orthopaedic Physical Therapy, Faculty of Physical Therapy, Nahda University in Beni Suef, Beni Suef, Egypt; ^2^Department of Orthopaedic Surgery, Faculty of Medicine, Cairo University, Giza, Egypt; ^3^Department of Anatomy and Embryology, Faculty of Medicine, Cairo University, Giza, Egypt; ^4^Department of Histology, Faculty of Medicine, Cairo University, Giza, Egypt

**Keywords:** double crush syndrome, median nerve, carpal tunnel syndrome, forearm, cervical radiculopathy

## Abstract

**Purpose:** This study aims to histologically compare the median nerve in the arm, forearm, and wrist, to help understand how cervical radiculopathy in a double crush phenomenon causes distal nerve dysfunction at the carpal tunnel and median nerve with concurrent absence of symptoms at the forearm.

**Methods:** The study was performed on 12 fresh cadaveric upper limbs free from any injury or operation. Male cadavers in the age range of 35–40 years were used. The dissection of the median nerve and the histological examination of the specimens from the arm, forearm, and wrist were conducted to evaluate variations in the epineurium thickness (μm), perineurium thickness (μm), number of fascicles per nerve trunk, area percent of myelin covering, and area percent of neurolemmal sheath.

**Results:** Morphometric and statistical results of the cadaveric median nerve trunk revealed that the mean epineurium and perineurium thickness measured in H&E-stained sections in the forearm were significantly greater than those in the arm and wrist specimens. Further, the mean percent area of the myelin covering in the forearm was significantly lower than that in the arm and wrist specimens in the sections stained with osmium oxide (*p* < 0.001). There were, however, no significant differences in the neurolemmal sheath among the arm, forearm, and wrist specimens in the silver-stained sections.

**Conclusion:** The histological differences explained the high concomitant occurrence of carpal tunnel syndrome (CTS) and cervical radiculopathy and the concurrent absence of symptoms at the forearm. Hence, we suggest cautious evaluation of patients with upper limb symptoms, since the management of these conditions requires a different approach.

## Introduction

The median nerve is a peripheral nerve and is one of the five terminal divisions of the brachial plexus. Further, the median nerve is formed by the convergence of the lateral and medial cords of the brachial plexus, and it has connections from all anterior rami of C5-T1 (1).

The median nerve is a mixed (sensory and motor) nerve. It is classically described as the nerve of pronation, of thumb, index finger, middle finger, and wrist flexion; of thumb antepulsion and opposition; as well as the nerve of sensation for the palmar aspect of the first three fingers. The median nerve is named so because of its middle position at the end of the brachial plexus and the forearm. During its course, from its origin at the brachial plexus to its terminal branches, the median nerve runs through various narrow passages where it could get compressed at the carpal tunnel, resulting in carpal tunnel syndrome (CTS) (2).

The median nerve is formed by two roots–medial and lateral. The medial root from the medial cord and the lateral root from the lateral cord from the brachial plexus converge at the anterior part of the axillary artery, as the median nerve trunk runs downward from the lateral side of the brachial artery to the end point of the coracobrachialis muscle. Median nerve covers the superficial surface or the deep surface of the brachial artery and turns to the inner side of the artery, going downward to the chelidon along with the blood vessel. The median nerve and blood vessels extend downward through the pronator teres muscle and the flexor digitorum superficialis tendon arch; they then travel downward along the median nerve at the forearm and reach the wrist along the part between the flexor digitorum superficialis muscle and flexor digitorum profundus muscle. The median nerve runs between the flexor carpi radialis muscle tendon and palmaris longus tendon and then travels through the carpal canal where it branches on the deep surface of the palmar fascia and extends through the palm (3).

Surface projection line for the median nerve in the upper arm is derived from the brachial artery pulse at the upper part of the medial bicipital sulcus to the nearly inner side of the midpoint between the medial and external epicondyles of the humerus. The projection line for the median nerve in the forearm can be obtained by extending the above projection line to the midpoint between the flexor carpi radialis muscle tendon and the palmaris longus tendon (4).

Peripheral nerves are composed of bundles of nerve fibers and surrounding connective tissue sheaths, including blood vessels. Each individual nerve fiber and the supporting Schwann cells are surrounded by a loose connective tissue called, the endoneurium. A bundle of nerve fibers is held by collagen fibrils in the connective tissue and form fascicles surrounded by a dense connective tissue called the perineurium. All nerve fascicles within a nerve trunk are completely ensheathed by a dense and irregular connective tissue called epineurium, which is the outermost layer of connective tissue sheaths (5). The intricate structure and the molecular organization of the human myelinated axon are important in determining the efficient conduction of nerve impulses (6). Schwann cells in the nerves distal to a crush or cut injury have properties, which include generation and maintenance of these cells, and their function in myelinophagy, the autophagic breakdown of redundant myelin in injured nerves, is also detected (7).

In 1973, Upton and McComas hypothesized about double crush syndrome (DCS) and reported that “a symptomatic compression at one site predisposed a peripheral nerve to increased susceptibility to impairment at distal location” (8). It is questionable why a similar referred pain is experienced by patients with carpal tunnel but not experienced by patients who have the median nerve damaged by radiculopathy in the forearm or by any external crush injuries (9).

A lot of debate exists among surgeons related to the existence of the DCS and the mechanisms that could be responsible for producing it (8). Although there exists a relationship between cervical radiculopathy and CTS, the etiology of the DCS and the physiology of the condition are still undetermined (10).

In one study, despite successful surgical treatment for CTS, symptoms did not disappear to a satisfactory level in about 25% of the cases (11). Cervical radiculopathy should not have an effect on distal sensory conduction tests and on distal myelin; most of the patients with CTS were found to have prolonged sensory latency and focal demyelination at electrophysiological investigations (12). Electrodiagnostic studies of median nerve mononeuropathy failed to meet the anatomical and pathophysiological requirements of the double crush hypothesis in the majority of patients (10). It is still unclear why cervical radiculopathy and CTS are observed together, and the underlying mechanism is still controversial. There are many proposed mechanisms associated with a primary nerve disorder, which can indeed predispose the nervous system to secondary nerve disorders, such as impaired axonal transport (8, 13), decreased intraneural microcirculation (14, 15), and alteration of nerve elasticity (16).

It is difficult to comprehend how cervical radiculopathy causes distal nerve dysfunction at the carpal tunnel and median nerve with no concurrent symptoms at the forearm based on our experience in managing cases of DCS and reading previous studies which are included as references (8, 9). Therefore, this study aims to histologically compare the median nerve in the arm, elbow, and wrist to see the differences among these three sites which may help us to understand how the cervical radiculopathy in double crush syndrome affects the median nerve at the carpal tunnel without producing similar symptoms at the forearm.

## Materials and Methods

A retrospective review of 350 patients with cervical radiculopathy revealed 279 patients with concomitant median nerve symptoms at carpal tunnel; however, none of them revealed symptoms at the forearm.

### Study Design

This study is an observational study that involves both anatomical dissection of the median nerve and histological description of the median nerve in the arm, Forearm, and wrist. Fresh human right cadaveric upper limbs from 12 cadavers with a normal cervical spine and upper limbs were dissected for describing the histology of the median nerve. The average age was 37.5 years (range, 35–40 years). The limbs were kept refrigerated, and the average refrigeration time was 8 h (range, 5.5–9 h). The median nerve in each of the upper limbs was dissected for histological data collection.

The study was conducted in accordance with the Declaration of Helsinki (17). The study design was approved by the institutional review board (number 380) and was registered at ClinicalTrials.gov (Identifier: NCT04324281). Informed consent was obtained before death for donating the bodies for medical research.

Exclusion criteria involved limbs that revealed local trauma, scars indicating altered nerve anatomy, cervical radiculopathy, anatomical evidence of upper limb pathologies, surgeries or injuries at any part or the whole length of the limb or even the cervical area, or any systemic disease affecting the nerve fiber. One researcher (EE) performed all the dissections for reducing any inter-operator variability.

### Median Nerve Dissection

In the supine position, with the arm abducted to 90°, the skin of the upper limb was incised longitudinally along the midline from the shoulder to the palm. The skin and subcutaneous tissues were excised to expose the median nerve, and care was taken to avoid any damage to the nerve.

Dissection of the median nerve was performed along its course from the forearm to the wrist. The following landmarks were determined for taking specimens from the median nerve: at the middle of the arm at the level of insertion of the coracobrachialis muscle (point 1), at the upper border of the pronator teres muscle in the cubital fossa at the elbow (point 2), and at the proximal border of the flexor retinaculum in the wrist (point 3).

The median nerve was dissected and excised in twelve cadaveric upper limbs at three levels (the arm, forearm, and wrist). Each segment from the three levels of the median nerve was 1 cm long. Each of the segments was divided into two parts, proximal and distal, which were subjected to histological evaluation procedures.

### Histological Evaluation

The proximal segment of each specimen was fixed in 10% buffered formalin solution for 24 h, dehydrated in ascending grades of ethanol and embedded in paraffin wax. Transverse and longitudinal sections of 5–7 μm were cut and subjected to H&E (18) and histochemical staining. The silver impregnation method was performed for demonstrating neurofibrils and neurolemmal sheath (19). The distal segments of the specimens were processed using osmic acid staining. The specimens were fixed in 4% paraformaldehyde for 1–2 h, transferred to 1% osmic acid for 3–4 days, embedded in paraffin, and cut at 5–7 μm (20).

### Morphometric Analysis

Morphometric analysis was conducted using an image analyzer software, Leica Qwin 500 C (Leica Microsystems Imaging Solutions Ltd., Cambridge, UK). The parameters that were measured include (a) the mean thickness of the epineurium and perineurium in the H&E-stained transverse sections of the median nerve in the arm, forearm, and wrist; (b) the mean number of fascicles per nerve trunk in 10 high-power fields in the H&E-stained transverse sections of the median nerve in the arm, forearm, and wrist; (c) the area percent of myelin sheath in osmium oxide-stained sections in 10 high-power fields; and (d) the area percentage of neurolemmal sheath in silver-stained sections in 10 high-power fields.

### Statistical Analysis

Statistical analysis was performed using SPSS statistical software version 19.0 (IBM, Armonk, NY, USA). All values for the median nerve were expressed as mean ± SD. ANOVA was followed by the Bonferroni *post-hoc* test for detecting significant differences in the group. A value of *p* < 0.05 was considered statistically significant.

## Results

Twelve median nerve specimens were histologically analyzed in the arm, forearm, and wrist for detecting variations in the epineurium thickness (μm), perineurium thickness (μm), number of fascicles per nerve trunk, area percent of myelin covering, and area percent of neurolemmal sheath (Table 1).

**Table 1:** Histological examinations of the median nerve in the arm, forearm, and wrist.

**Mean ± SD**	**Arm**	**Forearm**	**Wrist**
Epineurium thickness (μm)	3.3 ± 2.1	8.36 ± 3.53^*^	4.52 ± 2.07
Perineurium thickness (μm)	2.60 ± 2.42	6.08 ± 1.35^*^	2.25 ± 0.15
Number of fascicles per nerve trunk	9.50 ± 1.29	8.75 ± 1.71	8.38 ± 2.17
Area percent of myelin covering	5.28 ± 3.05	1.62 ± 1.05^#^	4.0 ± 2.40
Area percent of neurolemmal sheath	11.52 ± 2.13	12.95 ± 4.19	11.67 ± 3.03

* *significant increase compared to the arm group and wrist group;*

# *significant decrease compared to the arm group and wrist group*.

**Figure 1:**
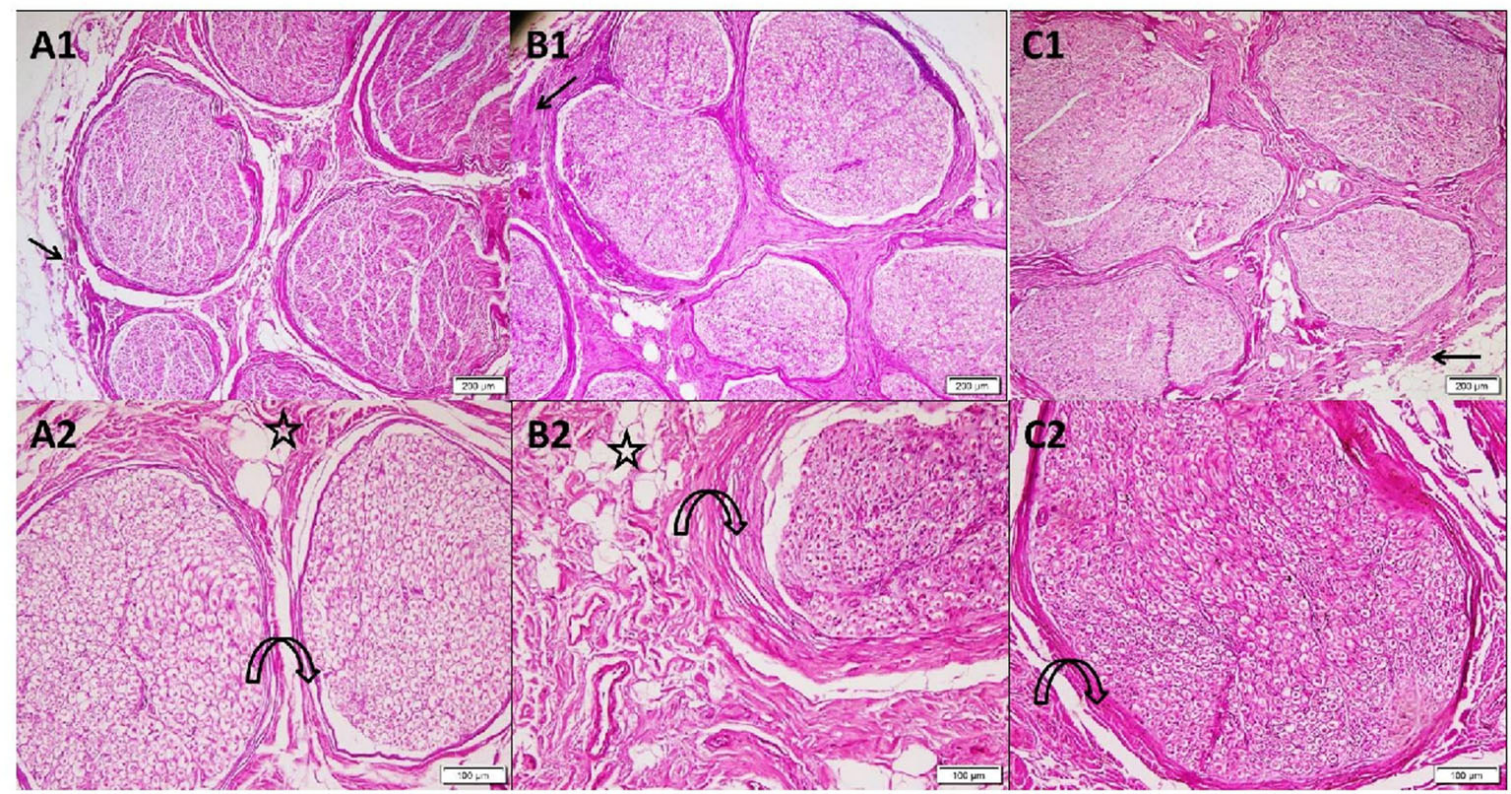
Photomicrographs of transverse sections in cadaveric median nerve (H&E). Group A: **(A1)** nerve trunk from the arm with its outer covering epineurium (arrow). The nerve consists of multiple nerve bundles surrounded by connective tissue perineurium (4); **(A2)** a higher magnification demonstrating fascicles with the surrounding perineurium (curved arrow) adipose tissue present in between the fascicles (star) (10). Group B: **(B1)** nerve trunk from the forearm showing multiple nerve bundles surrounded by thick connective tissue perineurium (arrow) (4); **(B2)** a higher magnification demonstrating bundle with adipose tissue (star) in the surrounding thickened perineurium (curved arrow) (10). Group C: **(C1)** nerve trunk from the wrist surrounded by epineurium (arrow). The nerve consists of multiple nerve bundles surrounded by the connective tissue perineurium (4); **(C2)** a higher magnification demonstrating nerve bundle with its surrounding perineurium (curved arrow) (10).

In group A, the sections stained with H&E of the cadaveric median nerve illustrated the nerve trunk from the arm with its outer covering, the epineurium. The nerve consisted of multiple nerve bundles surrounded by connective tissue perineurium with adipose tissue in between the fascicles. Group B nerve trunk from the forearm revealed multiple nerve bundles surrounded by thick connective tissue perineurium and adipose tissue. In group C, the nerve trunk from the wrist consisted of multiple nerve bundles surrounded by the perineurium (Figure 1).

**Figure 2:**
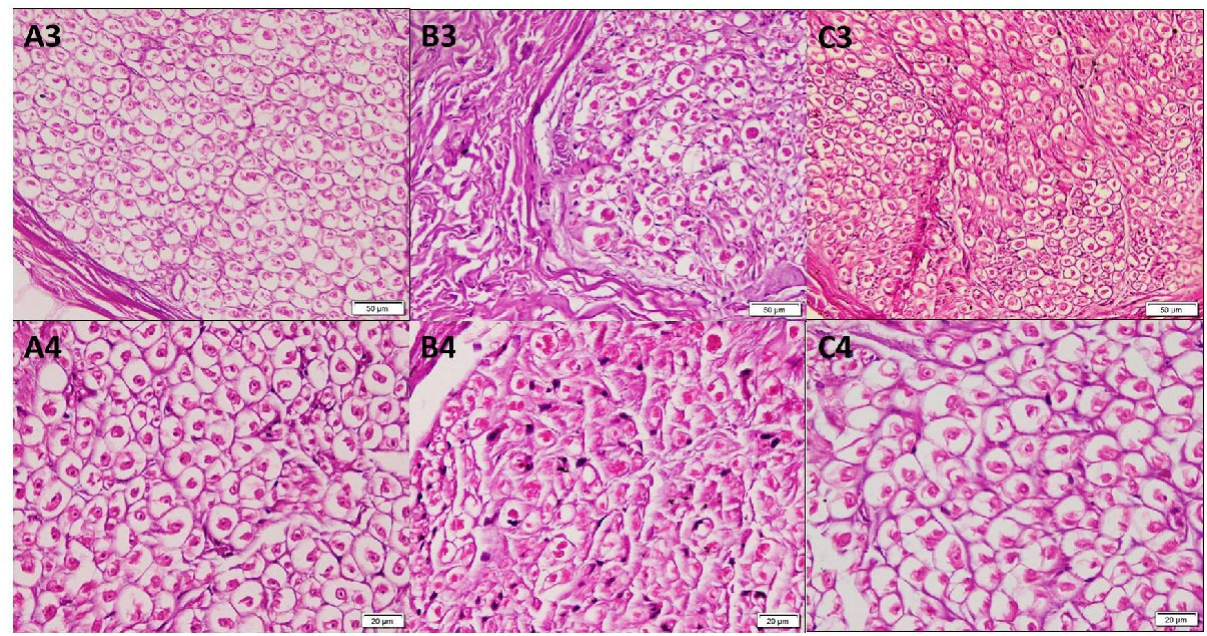
Photomicrographs of transverse sections in cadaveric median nerve (H&E). Group A: **(A3)** nerve trunk from the arm (20); **(A4)** a higher magnification demonstrating many acidophilic axons surrounded by the neurolemmal sheath endoneurium (40). Group B: **(B3)** nerve trunk from the forearm (20); **(B4)** a higher magnification demonstrating axons surrounded by the neurolemmal sheath endoneurium (40). Group C: **(C3)** nerve trunk from the wrist (20); **(C4)** a higher magnification (40).

The mean epineurium thickness (μm) in the forearm (8.36 ± 3.53) was significantly higher than that in the arm (3.3 ± 2.1) and wrist (4.52 ± 2.07) (*p* < 0.001). Higher magnification of the H&E-stained sections in group A and group C demonstrated many acidophilic axons surrounded by the neurolemmal sheath endoneurium. In group B, the nerve trunk from the forearm revealed small axons surrounded by the neurolemmal sheath (Figure 2). The mean perineurium thickness (μm) in the forearm (6.08 ± 1.35) was significantly higher than that in the arm (2.60 ± 0.42) and wrist (2.25 ± 0.15) (*p* < 0.001).

**Figure 3:**
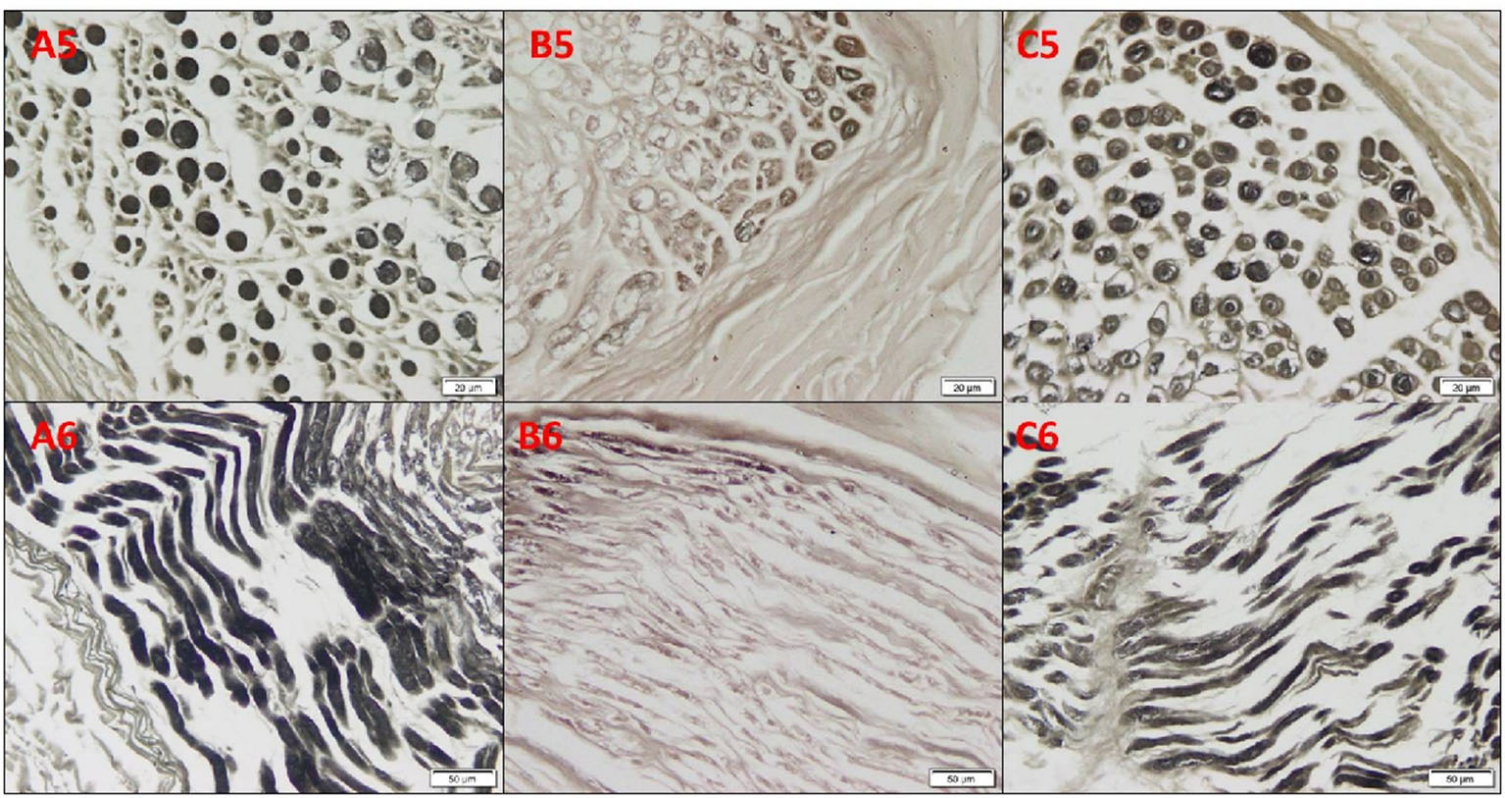
Photomicrographs of the cadaveric median nerve sections for demonstrating myelin sheath in transverse sections in **(A5, B5, C5)** and in longitudinal sections in **(A6, B6, C6)** of the group A arm, group B forearm, and group C wrist, respectively, revealing apparently normal myelin sheath stained black in the arm and wrist, and with a diminished staining in the forearm (osmium oxide, ×20).

**Figure 4:**
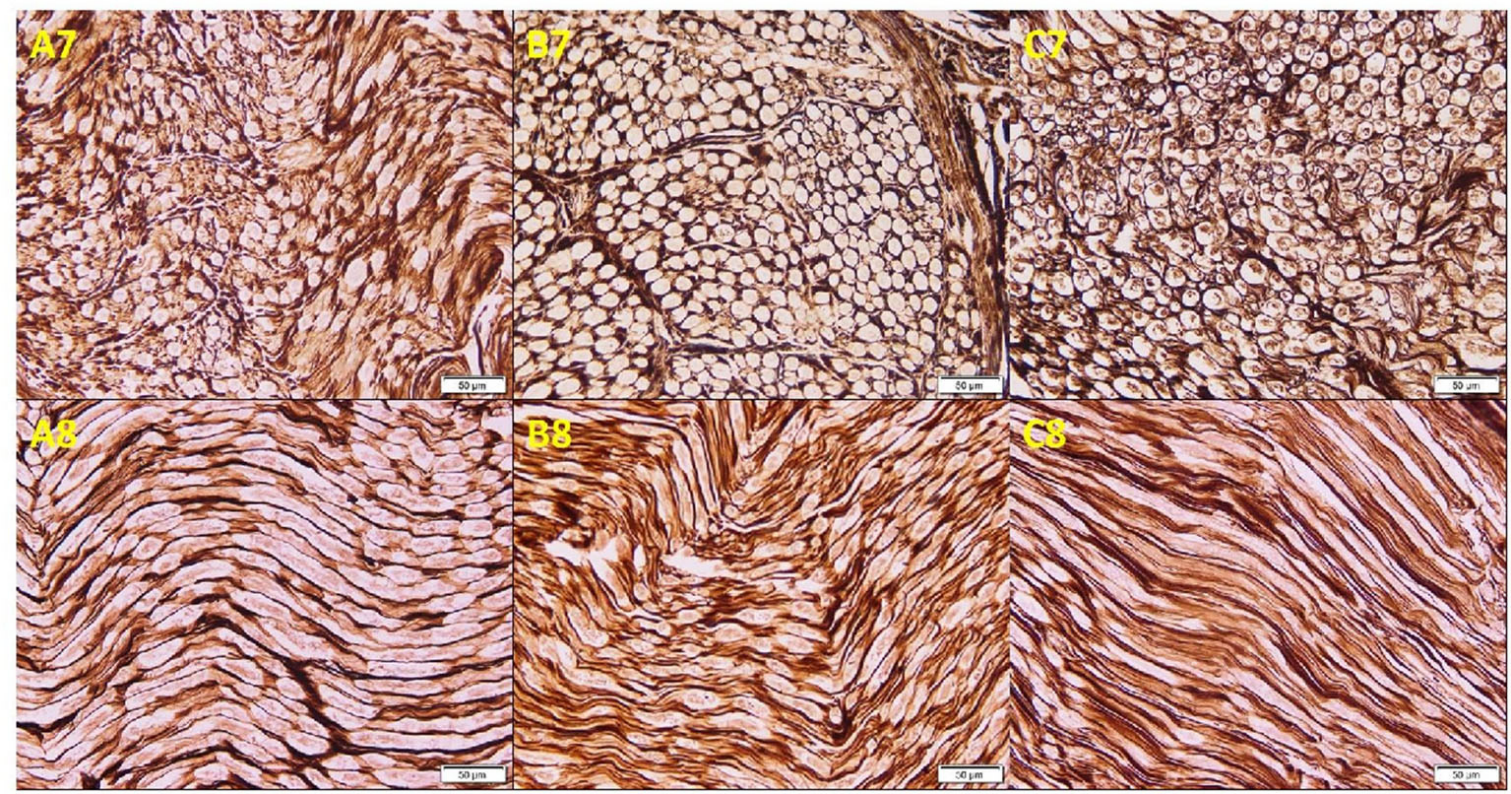
Photomicrographs of the cadaveric median nerve sections demonstrating transverse sections in **(A7, B7, C7)** and longitudinal sections in **(A8, B8, C8)** of groups A, B, and C, respectively, illustrating apparently normal neurofibrils, Schwann cell nuclei, and axons stained brown in the arm, forearm, and wrist (silver impregnation, ×20).

Osmium oxide stain results of the cadaveric median nerve transverse sections demonstrated apparently normal myelin sheath stained black in the arm and wrist, while a diminished stain was observed in the forearm (Figure 3). The mean number of fascicles per nerve trunk in the arm was 9.50 ± 1.29; forearm, 8.75 ± 1.71; and wrist, 8.38 ± 2.17, and no differences were detected. Silver impregnation results of the transverse sections of the cadaveric median nerve revealed apparently normal neurofibrils, Schwann cell nuclei, and axons stained brown in the arm, forearm, and wrist (Figure 4). The area percent of the myelin covering significantly decreased in the forearm, compared to the arm and wrist (1.62 ± 1.05, 5.28 ± 3.05, and 4.0 ± 2.40%, respectively) (*p* < 0.001). The area percent of the neurolemmal sheath in the arm, forearm, and wrist were 11.52 ± 2.13, 12.95 ± 4.19, and 11.67 ± 3.03%, respectively, without any significant differences.

## Discussion

This study aimed to histologically compare the median nerve in the arm, forearm, and wrist for investigating the occurrence of distal nerve disorder in DCS of the median nerve of the wrist. The study was performed on twelve fresh cadaveric upper limbs free from any injury or operation. This study investigated the histological differences in the median nerve in the arm, forearm, and wrist, and gave a comprehensive histological explanation for the secondary nerve disorder owing to the double crush of the median nerve appearing in the wrist and not the forearm. The results of this study suggest that cautious evaluation of the patients with upper limb symptoms is important because the management of these conditions is quite different.

In 1973, Upton and McComas (8) hypothesized that the axons that were compressed at one site become susceptible to damage at another site, and the hypothesis was based on the high prevalence (5–94%) of cervical radiculopathy in patients with CTS (21).

Several studies have been done on cervical radiculopathy owing to the poor surgical outcomes after carpal tunnel release, which could be due to the “double crush” phenomenon in which proximal nerve compression causes compression at the distal nerve, leading to distal nerve compression symptoms, which are not relieved owing to proximal pathology. Therefore, surgeons should pay close attention and investigate the involvement of proximal nerve pathology in patients with incomplete relief of symptoms after carpal tunnel release (22).

In addition to the debate on the presence of double crush, there is also a controversy regarding the mechanism that causes this phenomenon. Proposed mechanisms, which are associated with primary nerve disorder and might be a causative factor for secondary nerve disorders, are impaired axoplasmic flow in nerve fibers (8), reduced microcirculation (14), and altered nerve elasticity (23).

Cervical radiculopathy does not affect distal sensory conduction examination and distal myelination, but might have a little effect on the distal motor axon function of the median nerve, and the patients with CTS have sensory latency prolongation and focal dymelination (12).

It is difficult to understand the etiological relationship between cervical radiculopathy and CTS, since the anatomy and pathophysiology of the median nerve remain undetermined. In this study, the median nerve was dissected in 12 upper right arms of cadavers free from any pathology and with no history of any pathology at the cervical or whole arm or any systemic disease affecting nerve fibers. The specimens of the 12 median nerves from the arm, forearm, and wrist were compared histologically to identify why the elbow was not affected in the secondary nerve disorder.

Human peripheral nerves consist of three structural compartments: the outer epineurium, which consists of longitudinal arrays of collagen fibers that are important for maintaining structural integrity; the inner perineurium, which consists of concentric layers of specialized cells; and the innermost endoneurium, which consists of a looser mesh of collagen fibers. A nerve fascicle consists of the endoneurium and its surrounding perineurium (24). The mean thickness of the epineurium (8.36 ± 3.53 μm) in the forearm was significantly high. The epineurium is highly vascular, making it difficult to render any part of the peripheral nerve ischemic. The blood vessels are slightly coiled for adapting to the excursion of the nerves. Feeder vessels from the epineurial blood vessels course to all the inner parts of the nerve, and lymphatic vessels are found within the epineurium (25).

The epineurium that surrounds the median nerve is thicker at the forearm than in the arm and wrist, thereby protecting the nerve at the forearm from being crushed; providing elasticity and vascularity, and enabling better insulation and protection at the forearm. This explains why the elbow escapes injury while the wrist gets affected by the injury.

Furthermore, the mean perineurium thickness in the forearm (6.08 ± 1.35 μm) was significantly high. The intact perineurium protects the nerve and also hinders drug delivery to the nerve. Thus, the perineurium plays a critical role in nerve regeneration *via* the stimulation of perineurial glia cells (26) and is the major contributor to the nerve tensile strength (27). The perineurium appears thicker in the forearm than in the arm and wrist, thereby providing more protection against elongation under strain and providing a good barrier effect in ischemia and recovery. This demonstrates that the manifestation of dual nerve injury cannot affect the nerve in the elbow.

No significant difference was observed in the mean number of fascicles per nerve trunk in the arm (9.50 ± 1.29), forearm, (8.75 ± 1.71), and wrist (8.38 ± 2.17). Fascicles within the major peripheral nerve were divided repeatedly and fused to form fascicular plexuses; as the peripheral nerve divides and the number of fascicles decreases (24). This leads to nerve regeneration in the event of trauma.

The area percent of the myelin covering (1.62 ± 1.05%) was significantly lower in the forearm than in the arm (5.28 ± 3.05%) and wrist (4.0 ± 2.40%). Myelin sheath is a lipid–protein substance, which wraps axons and acts as an electrical insulator for facilitating electrical conduction in axons. Furthermore, large myelinated axons with large diameter conduct impulses more rapidly than small non-myelinated fibers (28). Therefore, the transmission of information is slower in the elbow than in the arm and wrist, which means that the effect on the nerve in the elbow will not be noticeable.

The area percent of the neurolemmal sheath at the elbow (12.95 ± 4.19%), arm (11.52 ± 2.13%), and wrist (11.52 ± 2.13%), showed no statistical difference. The neurolemma helps in the myelination and regeneration of the axons of the nerve. This occurs by the intact proximal neural fibrils growing into the distal neurolemmal tube (7). The median nerve in the forearm is histologically different with thicker epineurium and perineurium, which accounts for a more efficient blood–nerve barrier in the elbow. Further, the decrease in myelination leads to slow impulse transmission, which supports our work in this study.

The nerve bundles of the median nerve, at the level of the carpal tunnel, display no particular type of arrangement (29). The transligamentous course of the nerve is of special importance; it is usually accompanied by hypertrophic muscle, and the nerve hidden within this muscle can easily be cut during transection of the retinaculum. The results proved the necessity of approaching the median nerve from the ulnar side when opening the carpal tunnel (30).

The presence of a persistent median artery is an important consideration for plastic and orthopedic surgeons who frequently perform carpal tunnel release (31).

From the results of this study in understanding DCS, vascular pathologic factors contributing to compression pathology as explained in the histological examination should be considered. The median nerve does not give off any branches in the arm; it enters the forearm between the heads of the pronator teres supplying that muscle, palmaris longus, flexor carpi radialis, and flexor digitorum superficialis. It is normally according to the anatomy the patient have symptoms at forearm not at the wrist related to the median nerve pathway.

The electromyography of patients with carpal tunnel revealed slowing of nerve conduction proximal to the median nerve and failure at carpal tunnel release. This study might explain the occurrence of distal nerve disorder in DCS of the median nerve in the wrist.

The limitations of this study were the small number of cadavers. Further, the study was performed on only male cadavers.

In conclusion, the histological differences explained the high concomitant occurrence of CTS and cervical radiculopathy without concurrent symptoms at the forearm. Hence, we suggest cautious evaluation of patients with upper limb symptoms, which is important because the management of these conditions is quite different.

## Data Availability Statement

The data that support the findings of this study are available from the corresponding author upon reasonable request.

## Ethics Statement

The studies involving human participants were reviewed and approved by Approval Number 380 of faculty of Medicine Cairo University. The patients/participants provided their written informed consent to participate in this study.

## Author Contributions

SAA contributed to the conception and design of the study, experimental planning, acquisition of data, analysis and interpretation of data, statistical analysis, and drafting and revision of the manuscript. AF contributed to the conception and design of the study, experimental planning, acquisition of data, analysis and interpretation of data, statistical analysis, drafting and revision of the manuscript, funding acquisition, and management of resources. MA-W and EE-S contributed to experimental planning, acquisition of data, analysis and interpretation of data, statistical analysis, drafting, and revision of the manuscript. MM, SA, and SS-E contributed to the conception and design of the study, experimental planning, analysis and interpretation of data, statistical analysis, drafting and revision of the manuscript, funding acquisition, and management of resources. All the authors included in this manuscript made substantial contributions to this study, fulfilled the criteria for authorship, read, and approved the final manuscript.

## Conflict of Interest

The authors declare that the research was conducted in the absence of any commercial or financial relationships that could be construed as a potential conflict of interest.

## Publisher's Note

All claims expressed in this article are solely those of the authors and do not necessarily represent those of their affiliated organizations, or those of the publisher, the editors and the reviewers. Any product that may be evaluated in this article, or claim that may be made by its manufacturer, is not guaranteed or endorsed by the publisher.
